# Nemolizumab for treatment of lichen amyloidosis

**DOI:** 10.1016/j.jdcr.2025.06.010

**Published:** 2025-06-19

**Authors:** Seitaro Nakagawa, Ikuko Ueda-Hayakawa, Manabu Fujimoto

**Affiliations:** a Department of Dermatology, The University of Osaka Graduate School of Medicine, Suita, Osaka, Japan; b Department of Cutaneous Immunology and Microbiology, The University of Osaka Graduate School of Medicine, Suita, Osaka, Japan

**Keywords:** lichen amyloidosis, nemolizumab

## Introduction

Lichen amyloidosis (LA) is a subtype of primary localized cutaneous amyloidosis, clinically characterized by persistent pruritic, hyperkeratotic papules most commonly affecting the shins, forearms, and occasionally the trunk.^1^ Histologically, it is marked by the deposition of amyloid in the papillary dermis, usually secondary to chronic scratching. LA is often associated with intense pruritus, which significantly impairs patients' quality of life. Current treatment options, including topical corticosteroids, antihistamines, phototherapy, and systemic immunosuppressants, may not always provide satisfactory relief, and therapeutic outcomes are often suboptimal.

Recent advances in our understanding of the immunological pathways involved in pruritic skin disorders highlighted the role of interleukin (IL) 31 as a key pruritogenic cytokine.^2^ IL-31 is primarily produced by activated T helper 2 cells and signals through a heterodimeric receptor composed of IL-31 receptor alpha and oncostatin M receptor β, which are expressed on keratinocytes, immune cells, and cutaneous sensory neurons. Activation of this pathway leads to inflammation and transmission of itch signals to the central nervous system.

Nemolizumab, a humanized monoclonal antibody targeting the IL-31 receptor A, has demonstrated efficacy in reducing pruritus in patients with atopic dermatitis and prurigo nodularis.^3^^,^^4^ However, its role in treating LA has not been previously reported. Here, we present the first documented case of LA showing a dramatic response to nemolizumab after a 2-year course of therapy. This case suggests that IL-31 signaling may play a pivotal role in the pathogenesis of LA and that IL-31 blockade could represent a promising therapeutic strategy in managing this difficult-to-treat condition.

## Case presentation

A 39-year-old male presented with an 11-year history of intensely pruritic, hyperpigmented papules on the extremities, and parts of the trunk. His medical history included atopic dermatitis and allergic rhinitis, suggesting a predisposition to T helper 2–driven inflammation. Previous treatments, including topical corticosteroids, antihistamines, and phototherapy, provided only partial or transient relief.

On physical examination, numerous hyperkeratotic, brownish papules coalescing into plaques were observed, symmetrically distributed on the extremities and parts of the trunk. A skin biopsy was performed, revealing marked hyperkeratosis of the epidermis, thickening of the granular layer, and overall elongation of the rete ridges. In the papillary dermis, deposits of eosinophilic, amorphous material were observed. Direct Fast Scarlet staining showed orange staining in the papillary dermis, which exhibited a green birefringence under polarized light. Based on these findings, a diagnosis of LA was made.

**Fig 1:**
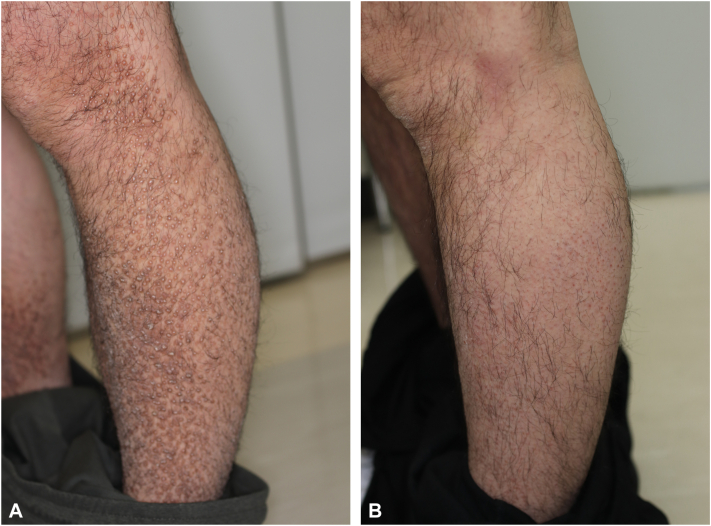
Hyperkeratotic, brownish papules coalescing into plaques were observed, symmetrically distributed on the extremities and parts of the trunk, which was biopsied and confirmed to be lichen amyloidosis. **A,** Lichen amyloidosis lesions before treatment. **B,** Lichen amyloidosis lesions mostly resolved after treatment.

Considering the inadequate response to conventional topical steroid treatment and the lack of effectiveness of excimer light phototherapy for more than 10 years and given the moderate severity of his concomitant atopic dermatitis, nemolizumab was initiated (60 mg subcutaneously every 4 weeks). Remarkably, pruritus had almost completely resolved within 6 weeks of treatment initiation. By 6 months, the lesions showed marked improvement, with substantial regression noted by the 12-month follow-up. At 24 months, the skin lesions had nearly completely resolved, leaving only mild residual elevation (Fig 1). No adverse effects were observed throughout the treatment period.

## Discussion

LA is a chronic pruritic disorder with limited effective treatments. IL-31 has emerged as a key contributor to its pathogenesis, particularly through its role in itch. ^5^^,^^6^ Nemolizumab, targeting the IL-31 receptor, directly interrupts this pathway and has shown promising results, supporting further exploration of IL-31 blockade in LA.

Dupilumab, which inhibits IL-4 and IL-13, has shown efficacy in LA, especially in cases with atopic features.^7^^,^^8^ Janus kinase (JAK) inhibitors, such as upadacitinib and abrocitinib, provide broader cytokine suppression and have been effective in refractory cases, offering rapid relief of itch and lesion improvement.^9^^,^^10^

In LA, chronic pruritus is the primary pathogenic trigger, directly driving scratching and amyloid deposition, whereas skin barrier dysfunction plays a secondary role.^5^ Unlike dupilumab or JAK inhibitors, which target broader type 2 inflammatory pathways, nemolizumab specifically blocks IL-31 signaling, the key driver of itch in LA. This targeted approach may offer more effective symptom control with fewer off-target effects, particularly in itch-dominant cases.

These findings suggest that IL-31–targeted therapy, such as nemolizumab, may be particularly well suited for LA, especially when pruritus is the dominant clinical feature like this case. While IL-4/IL-13 inhibition with dupilumab and broader JAK inhibition also show efficacy, their mechanisms may be more appropriate in cases with concomitant atopic inflammation. Collectively, these observations underscore the importance of prioritizing pruritus as a therapeutic target in LA. Further studies are warranted to evaluate the long-term efficacy and safety of these cytokine-targeted approaches.

In summary, this case represents the first documented successful treatment of LA with nemolizumab, highlighting its potential as a novel therapeutic option. Future research is needed to further elucidate IL-31's role in LA and its broader implications in dermatologic disease.

## Conflicts of interest

None disclosed.

## References

[1] Weyers W., Weyers I., Bonczkowitz M., Diaz-Cascajo C., Schill W.B. (1997). Lichen amyloidosus: a consequence of scratching. J Am Acad Dermatol.

[2] Bağci I.S., Ruzicka T. (2018). IL-31: a new key player in dermatology and beyond. J Allergy Clin Immunol.

[3] Kabashima K., Matsumura T., Komazaki H., Kawashima M., Nemolizumab-JP01 Study Group (2020). Trial of nemolizumab and topical agents for atopic dermatitis with pruritus. N Engl J Med.

[4] Kwatra S.G., Yosipovitch G., Legat F.J. (2023). Phase 3 trial of nemolizumab in patients with prurigo nodularis. N Engl J Med.

[5] Dousset L., Seneschal J., Boniface K. (2015). A Th2 cytokine interleukin-31 signature in a case of sporadic lichen amyloidosis. Acta Derm Venereol.

[6] Liu J., Chen J., Zhong Y. (2021). OSMRβ mutants enhance basal keratinocyte differentiation via inactivation of the STAT5/KLF7 axis in PLCA patients. Protein Cell.

[7] Zhu Q., Gao B.Q., Zhang J.F., Shi L.P., Zhang G.Q. (2023). Successful treatment of lichen amyloidosis coexisting with atopic dermatitis by dupilumab: four case reports. World J Clin Cases.

[8] Del Pozo D., Thampy D., Sun C.W., Hsu S. (2024). Use of dupilumab as a novel treatment for refractory lichen amyloidosis. JAAD Case Rep.

[9] Solimani F., Dilling A., Ghoreschi F.C., Nast A., Ghoreschi K., Meier K. (2023). Upadacitinib for treatment-resistant Lichen amyloidosis. J Eur Acad Dermatol Venereol.

[10] Zhang Y., Huang D., Gao Y. (2024). Case report: abrocitinib: a potential therapeutic option for lichen amyloidosis associated with atopic dermatitis. Front Immunol.

